# Vacuum-assisted closure combined with a closed suction irrigation system for treating postoperative wound infections following posterior spinal internal fixation

**DOI:** 10.1186/s13018-018-1024-6

**Published:** 2018-12-17

**Authors:** Kai Chen, Jin-ti Lin, Shuai-bo Sun, Jian Lin, Jian-zhong Kong, Nai-feng Tian

**Affiliations:** 1 Department of Spine Surgery, The Second Affiliated Hospital and Yuying Children’s Hospital of Wenzhou Medical University, Second Medical School of Wenzhou Medical University, Zhejiang Spine Surgery Centre, 109 Xueyuanxi Road, Wenzhou, 325027 Zhejiang China; 2 Department of Orthopaedic Surgery, The Second Affiliated Hospital and Yuying Children’s Hospital of Wenzhou Medical University, Second Medical School of Wenzhou Medical University, 109 Xueyuanxi Road, Wenzhou, 325027 Zhejiang China

**Keywords:** CSIS, Postoperative wound infection, Spinal surgery, VAC, Debridement, Wound healing

## Abstract

**Background:**

Wound infections after posterior spinal surgery are a troublesome complication; patients are occasionally forced to remove the internal fixation device, which can lead to instability of the spine and injury to the spinal cord. The purpose of this study was to evaluate the efficacy of modified vacuum-assisted closure (VAC) for treating an early postoperative spinal wound infection.

**Methods:**

We conducted a retrospective study of 18 patients with wound infections after posterior spinal surgery from 2014 to 2017 at a single tertiary center. All patients included in the study received modified VAC treatment (VAC combined with a closed suction irrigation system, CSIS) until the wound satisfied the secondary closure conditions. Detailed information was obtained from the medical records.

**Results:**

Wound size decreased significantly after 1 week of the modified VAC treatment. Three patients were treated with VAC three times and one patient received the VAC treatment four times; the remaining patients received the VAC treatment twice. The patients had excellent wound beds after an average of 8 days. The wound healed completely after an average of 17 days, and the average hospital stay was 33 days. There was no recurrence of infection at the 1-year follow-up.

**Conclusions:**

This study demonstrates that VAC combined with a CSIS is a safe, reliable, and effective method to treat a wound infection after spinal surgery. This improved VAC procedure provides an excellent wound bed to facilitate wound healing and shorten the hospital stay.

## Background

Various drugs, materials, and devices have been to treat postoperative wound infections. Vacuum-assisted closure (VAC) is a new method of wound nursing and treatment [1]. In the last decade, VAC, a product of modern medical progress, has reached new heights as a treatment for acute and subacute trauma, chronic wound infections, local tissue defects, and severe diabetic foot disease [2]. Nevertheless, the rate of use of VAC varies markedly among human body parts. To date, there have been few reports on the treatment of wound infections after posterior spinal internal fixation [3]. VAC is mainly used to treat lower extremity trauma with soft tissue defects and wound infections after internal fixation of lower extremity trauma [4–7]. The optimal procedure and treatment for wound infections after posterior spinal internal fixation are controversial. Active surgical procedures, including placement of drainage after repeated debridement and delayed closure, are advocated [8, 9]. The use of various medicinal ointments, including growth factors and enzyme debridement, can also influence on wound recovery. In 2010, Michael et al. reported on the use of a closed suction irrigation system (CSIS) to treat postoperative wound infections following posterior spinal fusion and instrumentation [10]. Traditional dressings have been used on surgical site infections for many years, but these tend to make patients extremely uncomfortable due to pain, foul smell, high cost, and prolonged hospital stay. These conditions have led to the development of a convenient, effective, and economic wound care method in which negative pressure is exerted on the wound after debridement. The use of negative pressure wound therapy is an innovative addition to the armamentarium of the spinal surgeon. Recent studies have shown that negative pressure wound therapy is a safe and effective technique in the treatment of wound infection after spinal surgery, and excellent clinical results have been obtained. A retrospective study of 30 cases has demonstrated that negative pressure wound therapy was safe in the treatment of wound infection after spinal internal fixation even with exposed dura [11].

Negative pressure wound therapy (NPWT), also known as VAC, involves the use of a polyurethane foam dressing, a transparent semi-permeable film, and a drain tube connected to a negative pressure bottle or a computer-controlled negative pressure pump. The VAC treatment principle is to create a vacuum environment, by applying continuous negative pressure to the surgical wound to remove the fluid between the tissue spaces and reduce edema [12]. In addition, continuous negative pressure attenuates internal fixation of bacteria, increases blood flow perfusion, and stimulates granulation and proliferation, thus providing good conditions for flap transplantation or secondary wound closure [13]. A randomized controlled study on the clinical efficacy of VAC for treating acute and chronic wounds showed that the method reduced wound size and length of hospital stay [14].

NPWT excludes tissue fluids from the wound site, but does not provide a perfectly clean granulation bed. Continuous saline lavage removes colonized bacteria, promotes the growth of granulation tissue, and provides a clean wound bed. Combination use of these two methods creates a better wound environment. A clean wound bed provides excellent conditions for secondary closure of the wound. VAC combined with a lavage system has been used for treating diabetic foot and mediastinitis, and the curative effect is remarkable [15–17]. Nine patients were treated with new continuous negative pressure and irrigation for infected wounds and intractable ulcers with excellent outcomes [18]. A recent study uses a well-controlled porcine model also confirmed that the new technique could better promote granulation response [19]. Much efforts have been made to improve the comfort of patients and reduce the need for repeated surgeries. However, no study has applied VAC with a CSIS to treat postoperative wound infection after spinal surgery. In this study, we retrospectively evaluated the efficacy of a modified VAC device (VAC combined with CSIS) to treat wound infections after spinal surgery.

## Materials and methods

### Patients

This was a single-group retrospective study carried out in the spine department at a single tertiary center. All patients signed informed consent forms prior to the study, which was approved by the Institutional Review Committee of our hospital and performed in accordance with the relevant regulations and guidelines.

All patients with a postoperative infection after spinal surgery between April 2014 and February 2017 were included in this study. A wound infection occurred in 29 patients after posterior spinal internal fixation. Patients with a superficial infection, malignant tumor, or malnutrition were excluded. Thus, 12 males and 6 females (age range, 35–80 years; mean age, 52 years) were included in this study. The preoperative diagnoses were thoracic/lumbar/sacral trauma and degenerative thoracic/lumbar disease. All patients were treated with intravenous prophylactic antibiotics after the initial treatment.

A wound infection after posterior spinal surgery was defined as (1) positive wound bacteria culture results and (2) signs and symptoms including red swelling and local pain at the surgical site, wound drainage (purulent exudation), wound dehiscence, and fever (> 38 °C).

### Treatment protocol

Patients with a wound infection after spinal surgery were treated with a traditional dressing during the early stage, which was changed once or more per day according to the wound exudate status. Drugs that promote wound healing, such as recombinant bovine basic fibroblast growth factor gel, were not used in this study. All patients subsequently underwent extensive debridement under anesthesia and received the modified VAC treatment. All internal fixation devices were retained during debridement. The key factors in debridement were removal of all necrotic and proliferating fibrous tissue, transplantation of allogeneic bone, and grafting of the margin.

The primary parameters evaluated included wound healing time, wound size, colonizing bacteria type, laboratory examinations, and hospital stay. The secondary outcome measures included granulation tissue, pain and discomfort, and treatment cost. Wound healing time was the interval between the first debridement and complete wound closure. Wound healing was defined as closure of the wound, without dehiscence, and with the surface of the wound being covered by epithelium and showing the ability to adapt to a certain level of tension and activity intensity. Calculation method of wound dimensions is as follows: after photocopying the size of the wound on the sterile paper, the electronic version of the image was obtained by the scanner, and the wound surface was eventually measured by standard 1-cm^2^ square drape. Laboratory examinations mainly included C-reactive protein, erythrocyte sedimentation rate, and leucocyte count. Detailed information was obtained from photographs and medical records. The main observation parameters were collected during debridement and then recorded and evaluated by professional recorders.

A single piece of polyurethane foam was cut to fit the shape of the wound and placed therein, with another foam dressing applied to the wound surface in patients treated with modified VAC. A flushing tube was inserted into the foam dressing, one end of which was placed in the wound gap. A transparent, semi-permeable film and drain tubes were placed on the wound surface simultaneously. A large volume of normal saline was injected into the wound through the flushing tube and exited through the exudate or necrotic tissue. The drain tube was subjected to a constant negative pressure (125 mmHg) for at least 1 week. The VAC dressing was changed twice weekly in all patients. For lavage fluid, 3000 ml of physiological saline was used and replaced once daily. The VAC treatment was stopped in all patients once the wound met the wound size closure conditions, and then, debridement and suturing were performed until the wound was completely healed. The wound secretions collected during debridement were cultured, and antibiotics were selected according to drug sensitivity test results. Vancomycin was used as an empirical antibiotic to treat the wound infections. Once the bacterial culture results were available, vancomycin was replaced by the antibiotic that the bacteria was sensitive to, and the treatment lasted for at least 2 weeks. Some patients took prophylactic antibiotics for 2 weeks or longer, as recommended by an infectious disease specialist.

### Statistical analysis

The statistical analysis was performed using SPSS software (ver. 20.0; SPSS Inc., Chicago, IL, USA). All quantitative variables are expressed as means ± standard deviation and *p* < 0.05 was taken to indicate statistical significance. Student’s *t* test was used to compare the wound parameters of patients treated with modified VAC.

## Results

Eighteen patients with a wound infection after posterior spinal surgery were included in the study; Table 1 shows their demographic characteristics. A laboratory examination revealed increased C-reactive protein (CRP) in 16 (88.9%) patients, an increase in the erythrocyte sedimentation rate (ESR) in 12 (66.6%) patients, and leukocytosis in six (33.3%) patients. All patients received treatment until wound healing was achieved. One of the patients developed a back rash during the VAC treatment, which was alleviated after taking antiallergic drugs. No other complications were observed during the modified VAC treatment. Another patient developed liver dysfunction after taking vancomycin, which was thus replaced by levofloxacin during subsequent treatment.

**Table 1:** Basic characteristics of patients

Characteristic	VAC (*n* = 18) (%)
Average age (year)	52
Range (year)	35–80
Male sex	12 (66.6)
Female sex	6 (33.3)
Risk factors	
Age > 60	6 (33.3)
Hypertension	4 (22.2)
Vascular disease	2 (11.1)
Diabetes	4 (22.2)
Smoking	1 (5.6)
Signs of infection	
Wound dehiscence	18 (100)
Fever	7 (38.9)
Wound pain	10 (55.6)
Wound discharge	18 (100)
Laboratory examination	
CRP > 8 mg/L	16 (88.9)
ESR > 20 mm/h	12 (66.6)
WBC > 10 × 10^9^ cells/ml	6 (33.3)

Data are no. (%) or mean (range)

*CRP* C-reactive protein, *ESR* erythrocyte sedimentation rate, *WBC* white blood cell

All patients were treated with prophylactic antibiotics before starting treatment; two were treated with clindamycin because of allergies. After the wound infection diagnosis, the antibiotics were changed according to the drug sensitivity test results. We obtained the wound change parameters from the medical records after 1 week of VAC treatment. The results show that the size of the wound after treatment with modified VAC was significantly smaller than that after debridement (*p* < 0.05). After 1 week of treatment, the length of the wound did not change significantly, but the width decreased from an average of 3.2 cm to 1.6 cm (*p* < 0.05). The average wound size was reduced from 23.5 to 13.2 cm^2^. Table 2 shows the patients’ initial diagnosis, treatment type, wound healing time, hospital stay, and treatment costs. The diagnosis of a surgical site infection was made 10.2 days after internal fixation. The average cost of a full course of VAC wound treatment was $1558.80. The total cost of the VAC dressing was significantly higher than that of a traditional dressing, but the time cost for clinicians and nursing staff was significantly lower for the VAC treatment than for the traditional dressing treatment. An excellent wound bed was achieved in all patients after an average of 8 days of VAC treatment. The patients were sent to the operating room to close the wound under anesthesia. Three patients were treated with VAC three times and one patient received VAC treatment four times, while the remainder received two VAC treatments. The average wound healing time and hospital stay of patients treated with modified VAC was 17 and 33 days, respectively.

**Table 2:** Wound and treatment characteristics of patients

ID	Age/sex	Diagnosis	Original operation^†^	Organism^‡^	No. of VAC changes	Onset time (days)	Healing time (days)	Treatment cost (USD)
1	44/M	T3–7 fracture	PFF	Se	2	10	15	1342.9
2	39/F	Sacral fracture	PDF	Ef	2	9	16	1333.4
3	70/M	Lumbar stenosis and spondylolysthesis	PDFF	MRSA	2	8	17	1472.4
4	67/M	T10 burst fracture	PDFF	–	3	6	20	1982.9
5	54/M	T11–12 fracture with dislocation	PDFF	Se	2	13	16	1380.5
6	62/F	Thoracic spinal stenosis	PDF	Ef	3	12	18	1862.5
7	36/F	T11 burst fracture	PF	–	3	6	19	1922.6
8	36/M	Thoracolumbar fracture (T9, L2)	PF	Se	2	8	16	1405.2
9	51/M	L1 burst fracture	PF	Sa	2	10	14	1410.6
10	45/M	L2, L4 fracture	PF	MRSA	2	8	15	1480.3
11	49/F	T12 burst fracture	PF	Sa	2	13	16	1520.8
12	65/M	T11, L1, L2 fracture	PDF	Se	2	14	15	1320.5
13	34/M	L1 burst fracture	PF	Ea	2	9	16	1385.6
14	53/F	T5, T6 fracture	PF	Ef	4	9	25	2601.9
15	80/M	T10 burst fracture, DISH^*^	PF	MRSA	2	12	18	1430.8
16	73/F	L1 burst fracture	PF	Sa	2	10	17	1300.9
17	35/M	L1 fracture with dislocation	PDFF	Pa	2	8	20	1560.2
18	54/M	Lumbar spinal stenosis	PDFF	–	2	18	16	1344.5

^*^DISH diffuse idiopathic skeletal hyperostosis

^†^*PFF* posterior fusion and fixation, *PDF* posterior decompression and fixation, *PDFF* posterior decompression and fusion + fixation

^‡^No bacteria or fungi exist in the culture, *Se Staphylococcus epidermidis*, *Ef Enterococcus faecalis*, *Sa Staphylococcus aureus*, *Ea Enterobacter aerogenes*, *Pa Pseudomonas aeruginosa*, *MRSA* methicillin-resistant *Staphylococcus aureus*

**Fig. 1:**
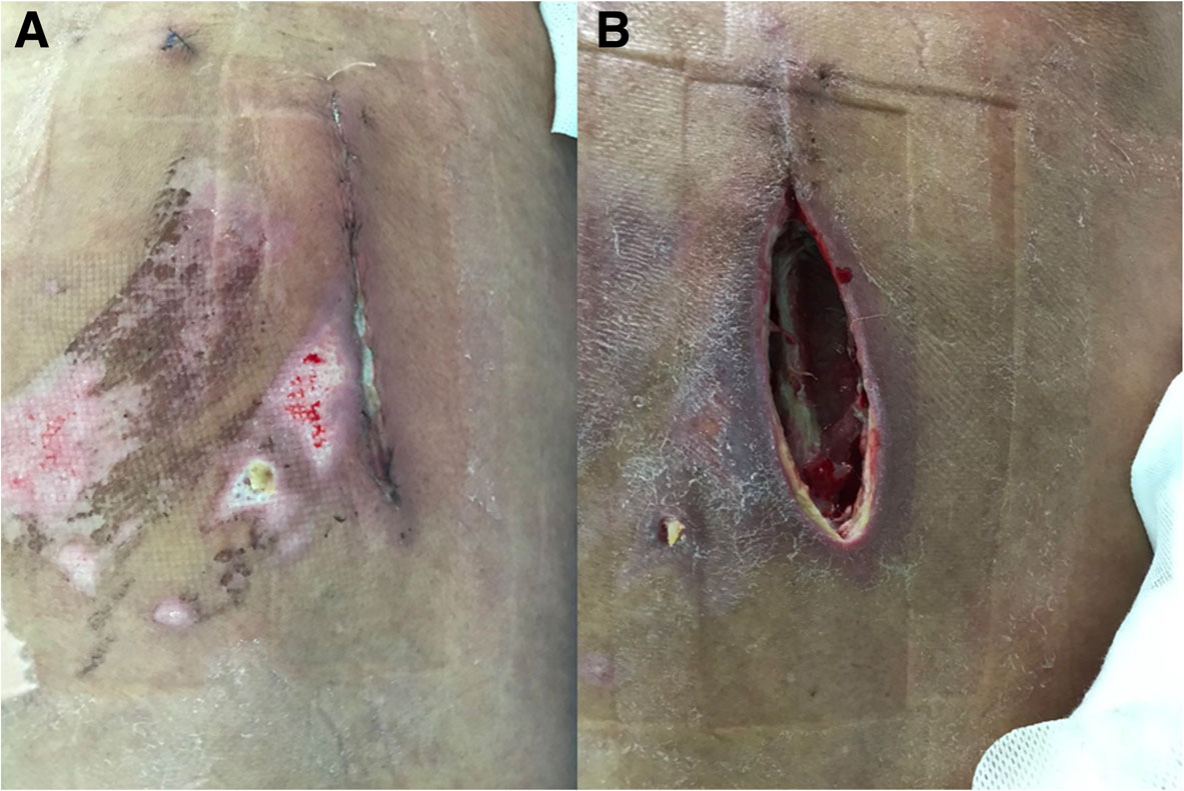
A typical case of modified VAC treatment: male, 67 years old. **a** Wound infection after posterior spinal internal fixation. **b** The wound after debridement

**Fig. 2:**
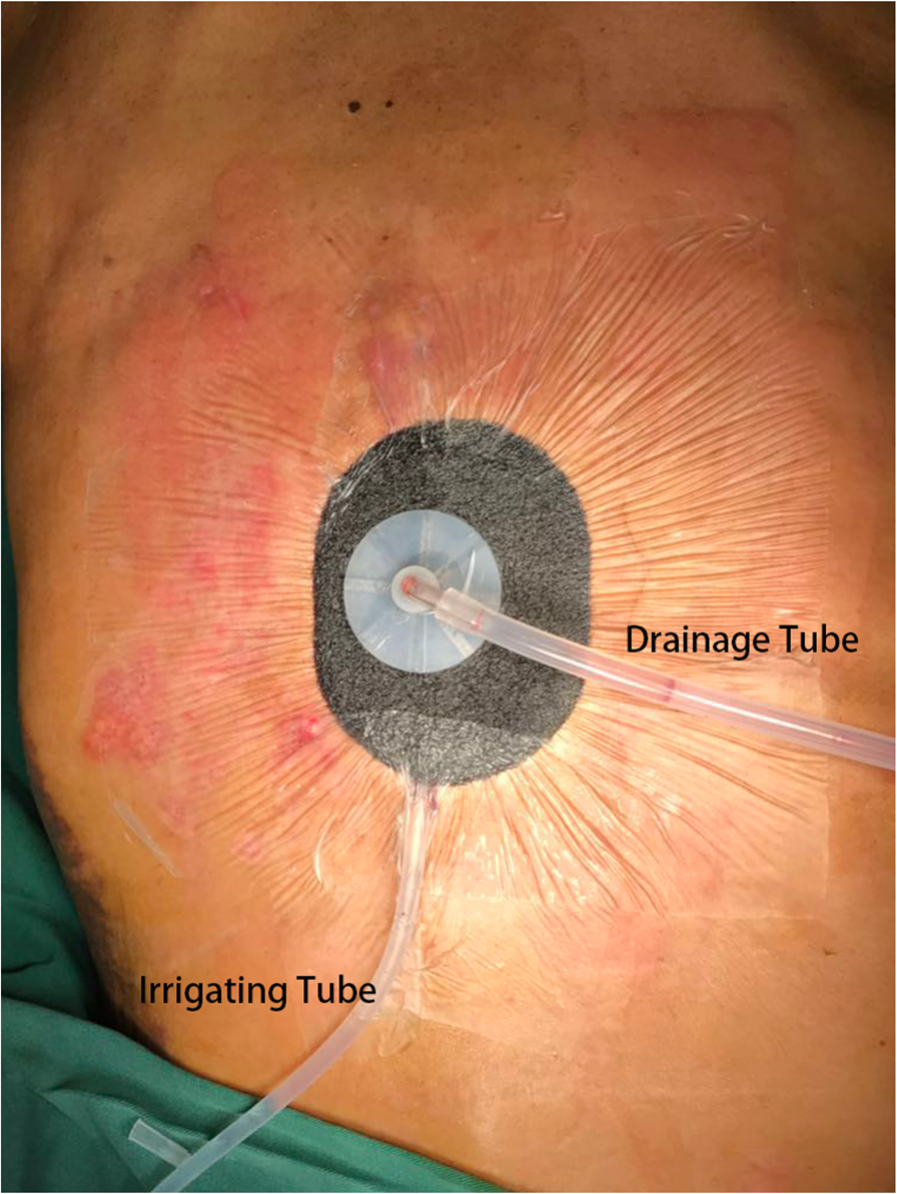
The application of VAC combined with closed suction irrigation system in the treatment of wound infection after posterior spinal internal fixation

**Fig. 3:**
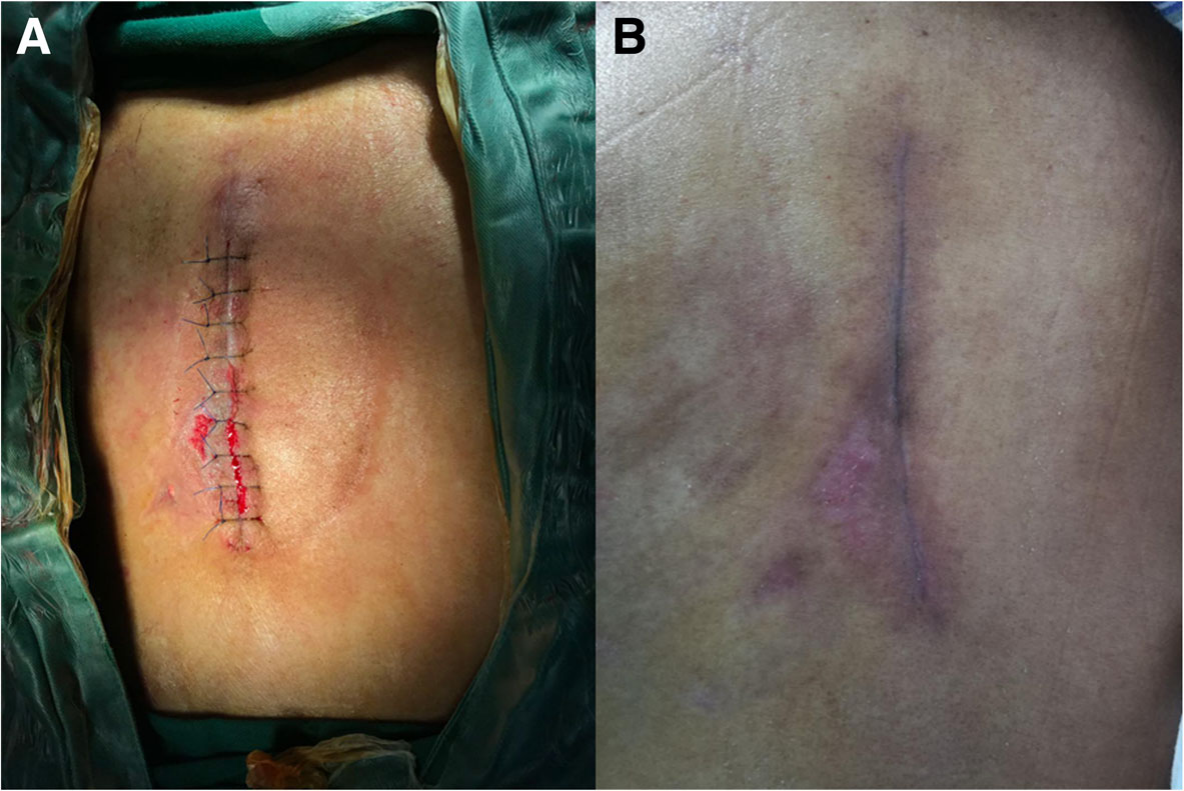
**a** Sutured wound. **b** The wound was completely healed

Wound secretions from 18 patients were cultured after debridement: there were six patients with *Staphylococcus aureus*, 4 with *Staphylococcus epidermidis*, 3 with *Enterobacter faecalis*, 1 with *Pseudomonas aeruginosa*, and 1with *Enterobacter aerogenes*; 3 patients had no bacteria in the wound (Table 3). There were seven cases with multidrug-resistant organisms, including three cases of *S. aureus*, three of *S. epidermidis*, and one of *P. aeruginosa*. Representative treatment cases are shown in Figs. 1, 2, and 3. All 18 patients retested laboratory examinations before leaving the hospital. The postoperative levels of leucocyte count were 6.03 ± 1.50 × 10^9^ cells/ml, and the erythrocyte sedimentation rate amounted to 15.72 ± 6.60 mm/h. The C-reactive protein level decreased to 6.88 ± 5.12 mg/L. All patients were followed-up for at least 1 year, and none of the patients developed a recurrent infection.

**Table 3:** Bacterial culture results

Pathogens	*N* = 18 (%)
*Staphylococcus aureus*	6 (33.3)
*Staphylococcus epidermidis*	4 (22.2)
Enterobacter faecalis	3 (16.7)
*Enterobacter aerogenes*	1 (5.6)
*Pseudomonas aeruginosa*	1 (5.5)
No bacteria	3 (16.7)

## Discussion

Wound infection after spinal surgery is a troublesome complication. Clinicians are attempting to resolve this problem using new methods with increasing frequency. Traditional dressings have been used to treat wound infections for a long time; although frequent dressing changes maintain a clean wound bed, the space for granulation tissue is limited. Patients treated with traditional dressings often complain of pain, a foul smell, and other types of discomfort. Positive debridement and wound drainage are the standard treatment methods, but significantly prolong the hospitalization time of patients.

Several studies have shown that VAC significantly reduces wound size, promotes growth of granulation tissue, and reduces bacterial colonization [20]. The VAC device has been widely used to treat acute and chronic wounds, diabetic foot, bedsores, and skin grafts, but has been used relatively less frequently in patients with postoperative wound infections after spinal surgery. There have been few studies on VAC to treat wound infections arising from posterior spinal surgery. Long-term negative pressure can cause injury to the spinal cord; however, recent studies have shown that the use of VAC in wounds with dura exposure does not cause complications, such as cerebrospinal fluid leakage or a spinal cord injury [11, 21].

Simple negative pressure attenuates tissue fluid buildup but does not yield a perfectly clean granulation bed. Continuous irrigation can reduce infection rates and decrease the likelihood of further surgery [10]. An animal experiment also showed that continuous irrigation dilutes and removes excess exudates and infectious materials, while providing a clean wound surface [19]. Delayed closure of the wound is contingent on the overall wound environment. At the same time, a good wound bed relieves pain and discomfort. In a retrospective study of 23 patients, Lian et al. reported that CSIS was a safe and effective treatment for deep infection after spinal fusion surgery [22]. However, there are certain disadvantages associated with CSIS. The wounds of patients treated with CSIS may remain moist and require frequent dressing changes. Early deep wound closure is not conducive to direct observation of granulation growth and secondary necrosis. The duration of the irrigation is based on the clinician’s experience and is usually maintained for 6–10 days. There has been no report of a VAC device applied in combination with a CSIS for postoperative wound infections after spinal surgery.

We conducted this retrospective study to evaluate the efficacy of modified VAC to treat early postoperative wound infections. The modified VAC system increased long-term saline irrigation, thus avoiding bacteria colonization, prolonging the replacement time of the VAC dressing, and reducing treatment costs. This study confirmed that VAC combined with a CSIS significantly reduced wound size and healing time. The changes in wound size after modified VAC therapy may be related to granulation growth and negative pressure closure. All patients were followed-up for at least 1 year without recurrence of their wound infection. Patients who received the modified VAC treatment did not show any deterioration of neurological ability or other serious complications during the entire course of treatment. The VAC device was purchased from CG BIO Co. Ltd. (Seongnam, Republic of Korea). The VAC unit is more expensive than traditional dressings, but such dressings need to be changed frequently and require more nursing time. A 1-week bed-rest treatment was acceptable to the patients, and the reduced nursing time requirement should significantly reduce the stress on clinical workers.

The application of antibiotics has always been a difficult point in the treatment of wound infection. It may be a wise choice to determine antibiotics based on the bacterial culture. But bacterial culture usually takes several days to produce results, and vancomycin as an empirical antibiotic for infection is used early in the treatment of surgical site infections; 4–6 weeks of antibiotic use is endorsed by many authors [23–25]. In our study, the duration of use of antibiotics was primarily based on this criterion. Laboratory examinations (white blood cell count, erythrocyte sedimentation rate, C-reactive protein return to normal level), wound conditions (including no redness, no exudation), and wound secretion bacterial culture were indicators of stopping the use of antibiotics.

Diabetes, cardiovascular disease, smoking, malnutrition, and multiple trauma may be risk factors for early wound healing. No attribution analysis was performed in this study. However, a randomized controlled trial demonstrated that VAC is effective for treating severe diabetic foot disease [2].

In this study, the VAC device combined with a CSIS effectively removed wound secretions and reduced bacterial colonization, promoted granulation growth, and shortened wound healing time and hospital stay. However, this improved VAC system also has minor disadvantages, such as the recorders being unable to observe changes in wounds during treatment. Furthermore, continuous negative pressure confined patients to the ward.

Our study had a few limitations. First, the sample size was relatively small. Another limitation was that the choice of subjects was drawn from a single medical center. The lack of sample size prevented us from conducting a randomized controlled trial. However, the small sample size and significant decrease in the incidence of post-spinal infection may be attributed to the use of antibiotics and aseptic technique. We could not confirm that VAC reduced bacterial colonization, although there have recently been detailed reports of this [26–28]. The duration of VAC treatment is determined by the surgeon’s clinical experience. Further research is needed to determine the optimal duration for VAC.

## Conclusions

VAC combined with a CSIS is a safe, reliable, and effective method for treating wound infections after spinal surgery. The improved VAC system used in this study maintained an excellent wound bed and avoided the need for frequent dressing changes. Therefore, the improved VAC device is a good method for treating wound infections after spinal surgery. This system could replace traditional dressings as the optimum treatment for postoperative wound infections.

## Abbreviations

CRP: C-Reactive protein
CSIS: Closed suction irrigation system
DISH: Diffuse idiopathic skeletal hyperostosis
Ea: *Enterobacter aerogenes*
Ef: *Enterococcus faecalis*
ESR: Erythrocyte sedimentation rate
MRSA: Methicillin-resistant *Staphylococcus aureus*
NPWT: Negative pressure wound therapy
Pa: *Pseudomonas aeruginosa*
PDF: Posterior decompression and fixation
PDFF: Posterior decompression and fusion + fixation
PFF: Posterior fusion and fixation
Sa: *Staphylococcus aureus*
Se: *Staphylococcus epidermidis*
VAC: Vacuum-assisted closure
WBC: White blood cell
